# VapC proteins from *Mycobacterium tuberculosis* share ribonuclease sequence specificity but differ in regulation and toxicity

**DOI:** 10.1371/journal.pone.0203412

**Published:** 2018-08-31

**Authors:** Abigail Sharrock, Alaine Ruthe, Emma S. V. Andrews, Vickery A. Arcus, Joanna L. Hicks

**Affiliations:** School of Science, University of Waikato, Hamilton, New Zealand; University of Rochester, UNITED STATES

## Abstract

The chromosome of *Mycobacterium tuberculosis* (Mtb) contains a large number of Type II toxin-antitoxin (TA) systems. The majority of these belong to the VapBC TA family, characterised by the VapC protein consisting of a PIN domain with four conserved acidic residues, and proposed ribonuclease activity. Characterisation of five VapC (VapC1, 19, 27, 29 and 39) proteins from various regions of the Mtb chromosome using a combination of pentaprobe RNA sequences and mass spectrometry revealed a shared ribonuclease sequence-specificity with a preference for UAGG sequences. The TA complex VapBC29 is auto-regulatory and interacts with inverted repeat sequences in the *vapBC29* promoter, whereas complexes VapBC1 and VapBC27 display no auto-regulatory properties. The difference in regulation could be due to the different properties of the VapB proteins, all of which belong to different VapB protein families. Regulation of the *vapBC29* operon is specific, no cross-talk among Type II TA systems was observed. VapC29 is bacteriostatic when expressed in *Mycobacterium smegmatis*, whereas VapC1 and VapC27 displayed no toxicity upon expression in *M*. *smegmatis*. The shared sequence specificity of the five VapC proteins characterised is intriguing, we propose that the differences observed in regulation and toxicity is the key to understanding the role of these TA systems in the growth and persistence of Mtb.

## Introduction

The most significant feature of *Mycobacterium tuberculosis* (Mtb) is its ability to persist in the most extreme environments. The bacterium has the ability to persist in a non-replicative, drug tolerant state surviving threats from the host immune system and antibiotic therapies to awaken later and cause disease [1, 2]. This ability is thought to account for the long treatment duration required for tuberculosis chemotherapy. The mechanisms that mediate this persistent state are very poorly understood, despite being one of the most important areas of mycobacterial research [3, 4].

Toxin-antitoxin (TA) loci were originally discovered as selfish genetic elements on plasmids, but their contemporary biological role is a subject of much debate. TA systems are bicistronic operons, identified by characteristic interactions of a stable toxin protein and its unstable inhibitor (the antitoxin). The operon, most often, regulates its own expression, but in the absence of its continued expression the unstable antitoxin is degraded, releasing the activity of the toxin. The toxin targets essential cellular processes including DNA replication, translation, cell wall synthesis and cell division leading to growth inhibition and in some cases cell death.

There are five TA families characterised to date: Type I, where the antitoxin is a small RNA that binds to the toxin mRNA thereby sequestering its expression; Type II, the largest TA family, where the antitoxin is a protein which binds to and inhibits the activity of the toxin; Type III, where the antitoxin is a small RNA which binds to the toxin protein inhibiting its activity; Type IV, where the antitoxin interferes with binding of the toxin to its target; and Type V, where a protein antitoxin inhibits the toxin by specifically cleaving its mRNA.

**Fig 1:**
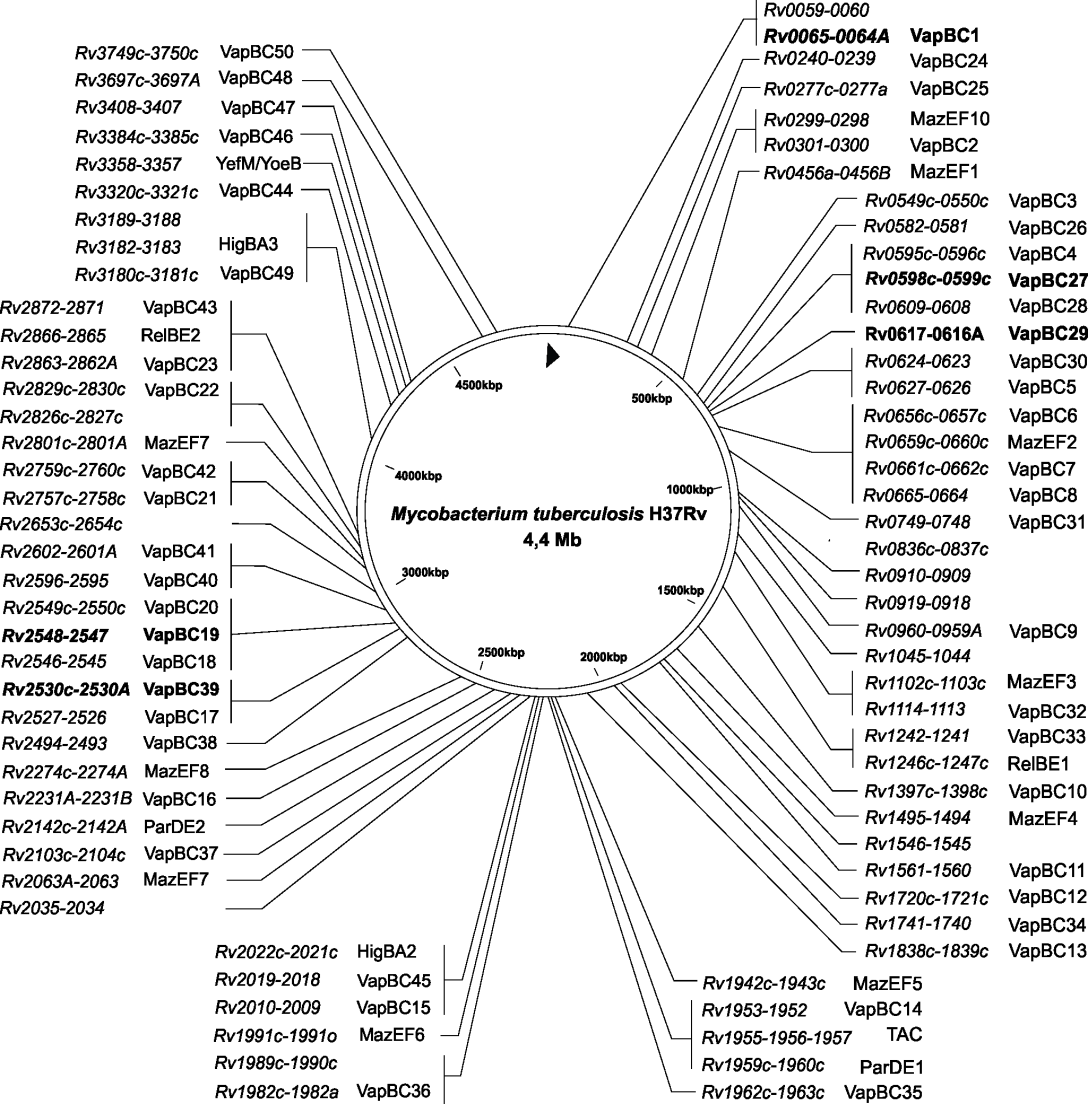
Location of toxin-antitoxin systems on the *M*. *tuberculosis* H37Rv chromosome. Most TA systems on this chromosomal map belong to Type II TA families with the exception of *Rv0836c-0837c*, *Rv1045-1044* and *Rv2826c-*2827c which are putative Type IV systems. VapBC systems investigated in this study are in bold text.

Compared to other non-pathogenic mycobacteria, Mtb has a greatly expanded number of Type II TA systems, which belong to the MazEF, RelBE, ParDE, HigBA and VapBC families (Fig 1) [5]. Of these, the most abundant is the VapBC family (Fig 1). Several of the Mtb VapBC loci are induced in response to stresses encountered during infection and invasion [6]. For example VapBC15, VapBC7 and VapBC25 are induced upon hypoxia and VapBC3, VapBC31 and VapBC49 were upregulated in drug-tolerant Mtb, suggestive of a role in persistence [7]. VapC from the non-pathogenic relative *Mycobacterium smegmatis* is a sequence-specific ribonuclease that cleaves mRNA transcripts to downregulate genes of sugar transport pathways, thereby coupling the rate of glycerol utilisation to bacterial growth [8]. To further support the role of VapC in regulating metabolism, the intra-cellular concentration of 11 VapC toxins from Mtb were found to be increased upon nutrient starvation [9].

The VapBC family is defined by the toxin (VapC) containing a PilT N-terminal (PIN) domain that has an RNase-H like fold and shows nuclease activity [10]. A negatively charged pocket is formed by four conserved acidic residues allowing for binding of a divalent metal cation thereby creating the active site for metal-dependent nuclease activity [11]. Of the 47 Mtb VapBC systems, four have been structurally characterised [12–15], and 21 out of 45 VapBCs tested were shown to be toxic in *M*. *smegmatis*, four of which were shown to inhibit translation [6]. Five VapCs shown to be toxic in *M*. *smegmatis* were bacteriostatic in Mtb [16].

Most VapC toxins tested to date show ribonuclease activity *in vitro*, including VapC1, VapC2, VapC5, VapC11, VapC20, VapC26 and VapC29 from Mtb [6, 12, 16–18], while VapC4 displays weak ribonuclease activity secondary to its RNA binding [19]. VapC from *M*. *smegmatis* cleaves mRNA in a sequence-specific manner [8] while two enteric VapC toxins from *Shigella flexneri* and *Salmonella enterica* cleave initiator tRNAs [20]. VapC20 from Mtb is a ribonuclease that specifically inhibits translation by cleavage of the Sarcin-Ricin loop of 23S rRNA [21]. Using a combination of UV-induced RNA-protein crosslinking and analysis of cDNA by high throughput sequencing, VapC proteins from Mtb (VapC4, VapC11, VapC37, VapC28 VapC15, VapC32, VapC25, VapC33 and VapC26) were shown to cleave RNAs essential for protein synthesis (tRNAs and rRNAs) [22]. Although VapC29 and VapC39 were shown to interact with tRNA7^Trp-CCA^ in sequencing experiments and subsequent northern analysis, no sequence-specific characterisation of the cut site was carried out [22]. We have previously characterised the sequence-specificities of both VapC1 and VapC29 from Mtb in an *in vitro* approach using pentaprobes and mass spectrometry [17]. Using an improved MALDI MS data analysis method we further characterise VapC1 and VapC29 along with VapC19, VapC27 and VapC39. Surprisingly, all five VapCs characterised demonstrate the same sequence-specificity, which led us to investigate the regulation and toxicity of these Mtb VapBC systems.

## Materials and methods

### Expression and purification of VapBC and VapC proteins in *Mycobacterium smegmatis*

VapBC complexes VapBC1 and VapBC29 were cloned into pYUB28b according to the method previously described in [23] and using primers listed in Table A in S1 File. The open reading frames (ORFs) encoding the *vapBC* operons VapBC27, VapBC39 and VapBC19 were amplified from *M tuberculosis* H37Ra genomic DNA using primers listed in Table A in S1 File. PCR products were digested using *Nco*I and *Hind*III restriction enzymes, purified, and inserted into the shuttle vector pYUB28b which introduced a C-terminal His-tag on VapC for downstream affinity purification. VapBC and VapC proteins were expressed in *M*. *smegmatis* and purified according to the method previously described [24], with the exception of VapC27 which instead required cation exchange purification post size-exclusion due to the higher isoelectric point of this protein.

### Ribonuclease activity assays

Ribonuclease activity assays were carried out as previously described [25]. In brief, assays to be analysed by urea-denaturing PAGE included purified VapC protein (2.9 μM), RNA (18.4 μM), 12 mM sodium phosphate buffer pH 7.4, 10 mM NaCl and 10 mM MgCl_2_ in a final volume of 10 μL. Individual assay reactions were set up for each time point to reduce the possibility of RNase contamination. Reactions were incubated for 5, 15, 30 or 60 minutes at 37°C. Reactions were stopped by the addition of 10 μL formamide loading dye and heating to 70°C for five minutes. Samples were run on 10–20% urea-denaturing gels (depending on the size of the RNA sample) to visualise RNase cleavage. A low range ssRNA ladder (New England Biolabs, USA) was run alongside samples in order to determine the sizes of bands. Gels were post-stained with SYBR^TM^ Green II RNA Stain (Thermofisher, USA) for 40 minutes and then visualised under UV light. For MALDI-TOF MS analysis, assays included VapC (2.9 μM), RNA (18 μM), 20 mM ammonium phosphate buffer pH 7.4, 20 mM NaCl and 10 mM MgCl_2_ in a final volume of 10 μl. Reactions were incubated for 5, 15, 30 or 60 minutes at 37°C. RNA was precipitated by ammonium acetate and ethanol precipitation ready for MALDI-TOF MS analysis.

### Determination of VapC cut sites

The mass list and corresponding intensities were exported from the MALDI-TOF analysis software into a comma separated excel file then processed using in-house software developed by Tony Smith and Vic Arcus (The University of Waikato) to determine possible cut sites. The software identifies all one point cuts in the oligonucleotide and calculates the putative mass for each resulting fragment, factoring in a 5′ phosphate on the 3′ cleavage product. Comparisons are made to the actual peak masses from the MALDI-TOF data, and those fragments resulting from single cuts which match the actual peak masses most closely are considered as putative VapC target cut sites. The intensity cut off was set to greater than 800, and as such, all peaks above an intensity of 800 were picked for analysis. Our improved in-house software for the analysis of RNA oligonucleotides focuses on identifying one point cut sites in their predominant orientation. This accounts for optimal cut sites, which would be cut preferentially over other cut sites almost immediately, producing just two fragments. The improved analysis also takes into consideration the intensity of the cleaved fragments of RNA, with predominant/optimal cut sites appearing with higher peak intensities compared to other cut sites. The previous analysis took into consideration all one and two point cut sites and every combination of their orientation without regards to peak intensity.

### Creation of DNA constructs for electrophoretic mobility shift assays

Promoter regions of the operons *vapBC1*, *vapBC27* and *vapBC29* were amplified using the primers listed in Table A in S1 File. For closer analysis of DNA-binding to the *vapBC29* promoter, shorter sequences (50 bp) were designed to span this promoter region. These were designed as single-stranded complementary DNA oligonucleotides which were to be annealed prior to use in binding assays (see Table A in S1 File). Oligonucleotides were made up to a final concentration of 100 μM in 1x TE buffer. Equal molar ratios of forward and reverse oligonucleotides were added to 10 μL Binding Buffer (50 mM Tris-HCl pH 8.0, 1 mM EDTA, 150 mM NaCl). Samples were heated at 95°C for 5 minutes and cooled to room temperature over 45 minutes. The single-stranded oligonucleotides and annealed product were run alongside each other on a 2% agarose gel to assess the success of the annealing reaction. DNA was labelled at the 3’ end with Digoxigenin (DIG)-11-ddUTP, as per the protocol outlined in the DIG Gel Shift Kit, 2^nd^ Generation (Roche).

### Electrophoretic mobility shift assays

EMSA reactions were set up on ice to a final volume of 6.5 μL. Into each reaction was added the appropriate volume of purified VapBC protein, 0.4 ng DIG-labelled DNA, 2 μl 5x Binding Buffer (Roche), 0.5 μl 1 mg.mL^-1^ poly dI-dC (Roche) and ultrapure water sufficient to adjust the total volume to 6.5 μl. Binding reactions were incubated for 20 minutes at room temperature. Reactions were placed back on ice and 2.5 μl loading buffer with bromophenol blue (Roche) was added. Tris-borate-EDTA gels (6%) were pre-run in 1 x TBE buffer at 60 V for 20 minutes. Samples were loading and run at 150 V until the dye-front had migrated approximately two-thirds of the way down the gel. Contact blotting was performed for 30 minutes using Whatman 2 MM blotting paper, after which the DNA was crosslinked to a Hybond-N^+^ (GE Healthcare) membrane using a Bio-Link BLX-254 BRL UV Crosslinker Irradiation System (Life Technologies) which irradiated the membrane with 120 mJ at UV 254 nm for 20 seconds. DNA was detected by a chemiluminescent immunoassay and visualised on films.

### Conditional expression of VapC and VapBC in *Mycobacterium smegmatis*

To create tetracycline inducible expression constructs of *vapBC* and *vapC* genes, the genes for operons *vapBC1*, *vapBC27* and v*apBC29* were amplified from protein expression plasmids (constructed in the previous section) using primers listed in Table A in S1 File. The PCR products were digested and ligated between the *Bam*HI and *Spe*I sites of the tetracycline-inducible vector pMIND and were transformed into *E*. *coli* TOP10 cells for selection on low salt LB medium supplemented with 50 μg.mL^-1^ hygromycin B. Sequencing confirmed the correct integration of the insert. Each of the forward primers introduced a synthetic ribosome binding site (RBS) upstream of the *vapBC* operon to ensure translation. Sequenced constructs as well as the intact empty pMIND vector (Empty Vector, EV) were transformed into *M*. *smegmatis* mc^2^155 and mc^2^155 *ΔvapBC* strains. For overexpression analysis, all *M*. *smegmatis* strains carrying pMIND vectors were grown to an OD_600_ between 0.2–0.4 in Luria-Bertani 0.05% (v/v) Tween-80 (LBT) broth supplemented with 50 μg.mL^-1^ hygromycin B. Each LBT starter culture was used to seed a second starter culture in Hartman's-de Bont minimal medium (HdB) supplemented with 0.2% glycerol, 50 mM MES, 0.05% (v/v) Tween-80 and 50 μg.mL^-1^ hygromycin B. HdB cultures were grown to an OD_600_ between 0.1–0.2 and diluted to an OD_600_ of 0.0025 in 200 mL HdB medium in a 500 mL flask. The growth of all strains was monitored until an OD_600_ of 0.1–0.15 was reached, after which protein expression was induced with 20 ng.mL^-1^ tetracycline (Tc) which had been previously reported to be sufficient for induction from the tetracycline-inducible promoter with limited toxicity to the cells [26]. Samples of culture were taken periodically to monitor the growth (OD_600_) of the culture and cell viability (CFUs). For cell viability, samples of culture were serially diluted 100-fold from 10^−2^ to 10^−6^ in 1x phosphate buffered saline (PBS) and each dilution was spotted onto plates supplemented with hygromycin B and tetracycline (+Tc), and hygromycin B only (-Tc).

## Results and discussion

### Description of *vapBC* operons tested

The 47 *vapBC* TA operons from Mtb were PCR amplified and cloned into the mycobacterial expression vector pYUB28b [27] to express the VapBC complex in *M*. *smegmatis* with a C-terminal His-Tag. Unannotated *vapB* genes upstream of an annotated *vapC* were identified by BLAST. Seventeen of the 47 VapBC complexes tested were expressed as soluble proteins in small scale expression trials (Table B in S1 File).

For this study, five VapBC systems (VapBC1, VapBC19, VapBC27, VapBC29 and VapBC39) were selected based upon: a range of positions on the chromosome (Fig 1), different VapB protein families (Table 1), and a high yield upon purification. Of these five VapBC systems, antitoxins VapB19 and VapB39 have RHH/CopG domains, VapB27 an AbrB/MazE domain and the remaining two VapB antitoxins contain no conserved domains (Table 1). Based on gel filtration chromatography the stoichiometry of the five VapBC complexes varied; all were multimers, exhibiting between three and five VapBC heterodimers per complex (Table 1 and Figure A in S1 File).

**Table 1:** VapBC systems characterised in this study. Conserved domains and homologous VapB proteins were identified by homology searches using pBLAST. Predicted molecular weights (MWs) of VapBC heterodimers were calculated by ProtParam [28]. Molecular weights of VapBC complexes were calculated by calibration of an S200 analytical gel filtration column (SEC). Stoichiometry of the VapBC complex was determined by division of the molecular weight from gel filtration analysis with the predicted molecular weight of a VapBC heterodimer.

VapBC operon	Genes *vapB/vapC*	VapB family	Homologous VapB	Predicted MW of VapBC heterodimer	Molecular weight (SEC)	Stoichiometry
VapBC1	Rv0064a/Rv0065	No conserved domains	Weakly similar to Rv0300 (VapB2) and Rv1721c (VapB12)	24.5	138	5.6
VapBC19	Rv2547/Rv2548	RHH, CopG family Pfam01402	37.1% identity to Rv1398c (VapB10)	27.33	99	3.61
VapBC27	Rv0599c/Rv0598c	AbrB, MazE superfamily COG2002	36% identity to Rv25395 (VapB40)	25.8	65	2.55
VapBC29	Rv0616a/Rv0617	No conserved domains	36% identity to Rv2530a (VapB39)	23.7	121	5
VapBC39	Rv2530a/Rv2530c	RHH [29]	56.5% identity to Rv2493 (VapB38)	24.20	119	4.9

VapBC complexes underwent a limited tryptic digest as in [8, 17] to remove the protease-susceptible VapB. VapC was subsequently purified by anion-exchange chromatography (Figure B in S1 File) and analysed by MALDI-TOF mass spectrometry and SDS-PAGE, to check the protein was intact and of the correct molecular weight (not digested by trypsin). Isolated VapC was then tested for ribonuclease activity.

### VapC proteins from *Mycobacterium tuberculosis* show the same sequence-specificity

**Fig 2:**
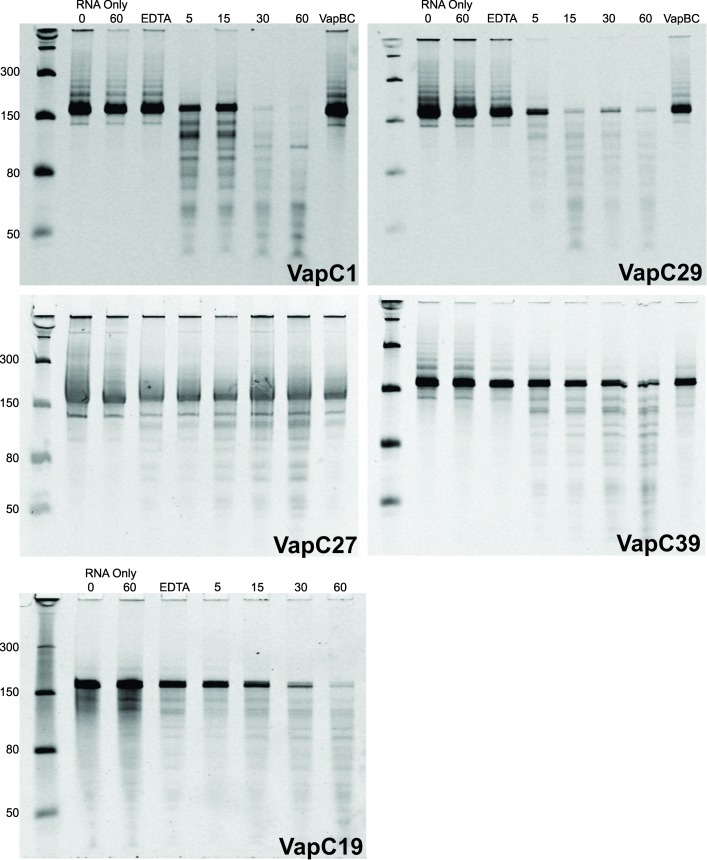
Five Mtb VapC proteins display the same sequence specificity against 924 Pentaprobe RNA. The ribonuclease activity of VapC1, 19, 27, 29 & 39 (2.9 μM) was tested against the 924 pentaprobe RNA (18 μM). Negative controls included no VapC (RNA only) at the beginning (RNA only 0) and end (RNA only 60) of the assay reaction, addition of EDTA, and addition of the VapBC complex instead of VapC (VapBC). Time points were taken at 5, 15, 30 and 60 minutes post incubation with VapC at 37°C. Assay reactions were analysed on 10% urea-denaturing PAGE gels.

Pentaprobes are a series of plasmids whose 160–200 bp inserts cover every combination of five base pairs [30]. Using *in vitro* RNA transcription, a series of mRNA transcripts can be made using each pentaprobe plasmid (922–927 and the complement strand 928–933) as a template. Using a combination of pentaprobe mRNA transcripts and MALDI-TOF mass spectrometry (MALDI-TOF MS) we previously reported magnesium-dependent, sequence-specific ribonuclease activity of VapC1 and VapC29 against two single-stranded RNAs [17]. VapC19, VapC27 and VapC39 were tested for activity using pentaprobe RNAs, and further pentaprobe RNAs were tested for VapC1 and VapC29. All VapC proteins exhibited sequence-specific ribonuclease activity against all pentaprobes tested after a minimum five minute incubation, with some pentaprobes cleaved more efficiently than others (Fig 2). Activity was inhibited when VapC was in complex with VapB, as well as with the addition of EDTA, confirming the catalytic mechanism of VapC is metal-dependent (Fig 2).

The cleavage patterns of the pentaprobe RNAs incubated with the three newly characterised VapC proteins were identical to each other, and also to those generated by VapC1 and VapC29 proteins (Fig 2). There is a difference in activity amongst VapC proteins, with VapC1 and VapC29 exhibiting more rapid degradation of RNA over 60 minutes compared to VapC27, 39 and 19 (Fig 2). We have previously used this system to characterise the VapC ribonuclease sequence-specificity of VapC proteins from *M*. *smegmatis* [8] and *Pyrobaculum aerophilum* [17]. The sequence specificity of VapC from *M*. *smegmatis* [8] differed from that of the two *P*. *aerophilum* VapC proteins [17] (which share the same sequence specificity) which again differ from that of the Mtb VapC proteins characterised here. Therefore, the presumed shared sequence specificity of the Mtb VapC proteins characterised is not an artefact of the pentaprobe system used in this study.

**Fig 3:**
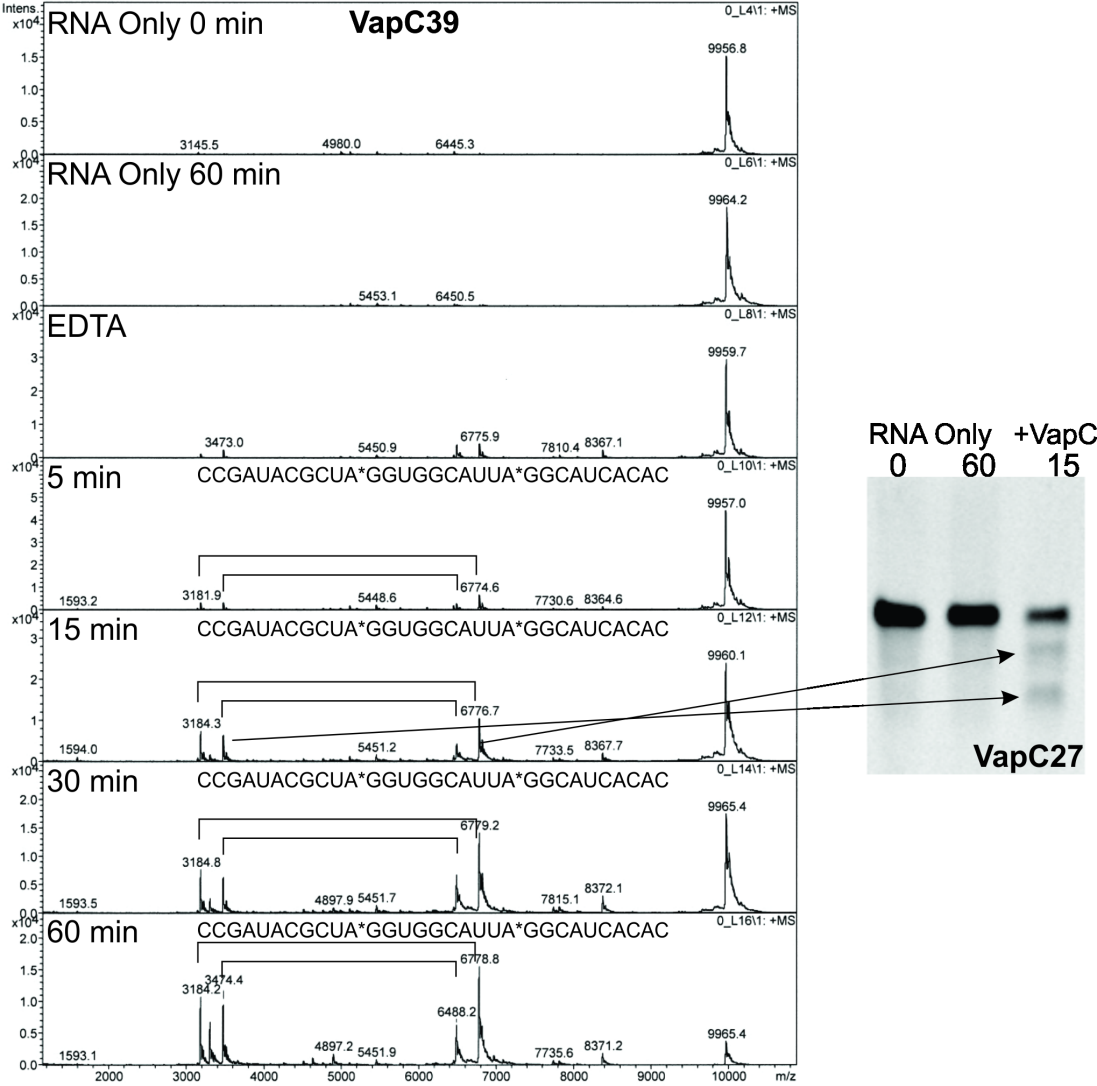
Determination of VapC sequence-specificity using MALDI-TOF MS and urea-denaturing PAGE. 932 RNA Oligo 5 VapC39 MALDI-TOF MS results. RNA Only 0 min and 60 min refer to negative control assay reactions with no VapC protein; 5, 15, 30 and 60 min represent a time course assay with VapC39. Data analysed by Bruker Data Analysis. The side panel shows 932 RNA Oligo 5 VapC27 urea-denaturing PAGE reuslts. R 0 hr and R 1 hr refer to negative control assay reactions with no VapC protein; 15 min represents assay time point with VapC27.

To confirm that the sequence specificity is indeed the same, VapC27 and VapC39 ribonuclease activity was assayed against nine RNA oligonucleotides spanning the 932 pentaprobe RNA. These oligonucleotides were readily available, having been used for the characterisation of VapC from *M*. *smegmatis* [8]. The cleaved fragments were analysed by urea-denaturing PAGE and MALDI-TOF MS (Fig 3).

We have previously reported that VapC1 and VapC29 target GC-rich 4-mer sequences in RNA [17]. We have since improved our in-house software for the analysis of RNA oligonucleotides which focuses on identifying one point cut sites in their predominant orientation (instead of both orientations) and the peak intensity of the fragments of RNA. This accounts for optimal cut sites, which would be cut preferentially over other cut sites almost immediately, producing just two fragments, at higher intensities compared to other cut sites. The previous analysis took into consideration all one and two point cut sites and every combination of their orientation and did not consider peak intensity of cut RNA fragments.

VapC39 932 oligo assays were analysed by MALDI-TOF MS using the improved data analysis method (Table 2 and mass spectra shown in Figures C—E in S1 File). The resulting peaks in the mass spectra were identical to VapC1 and VapC29 [25] (Figure F in S1 File) and a UA*GG optimal cut site was identified (Table 2 and Fig 3). VapC27 932 oligo assays were analysed by urea-denaturing PAGE. The RNA banding patterns observed on the gels reflect the masses seen in the VapC39 MALDI-TOF MS spectra for corresponding oligonucleotides (Fig 3). Together with the observation that VapC27 pentaprobe banding patterns are identical to those produced by VapC39 (Fig 2), we hypothesise an optimal cut site of UA*GG for VapC27 also. Re-analysis of the previous MS spectra collected for VapC1 and VapC29 using the new analysis method assigned major peaks to products of cleavage at UA*GG sites with a variety of secondary cut sites (mass spectra for RNA oligo 5 in Figure F in S1 File). The cut site UA*GG is present in Fig 5C in [17] but was masked by the large number of secondary cut sites identified.

**Fig 5:**
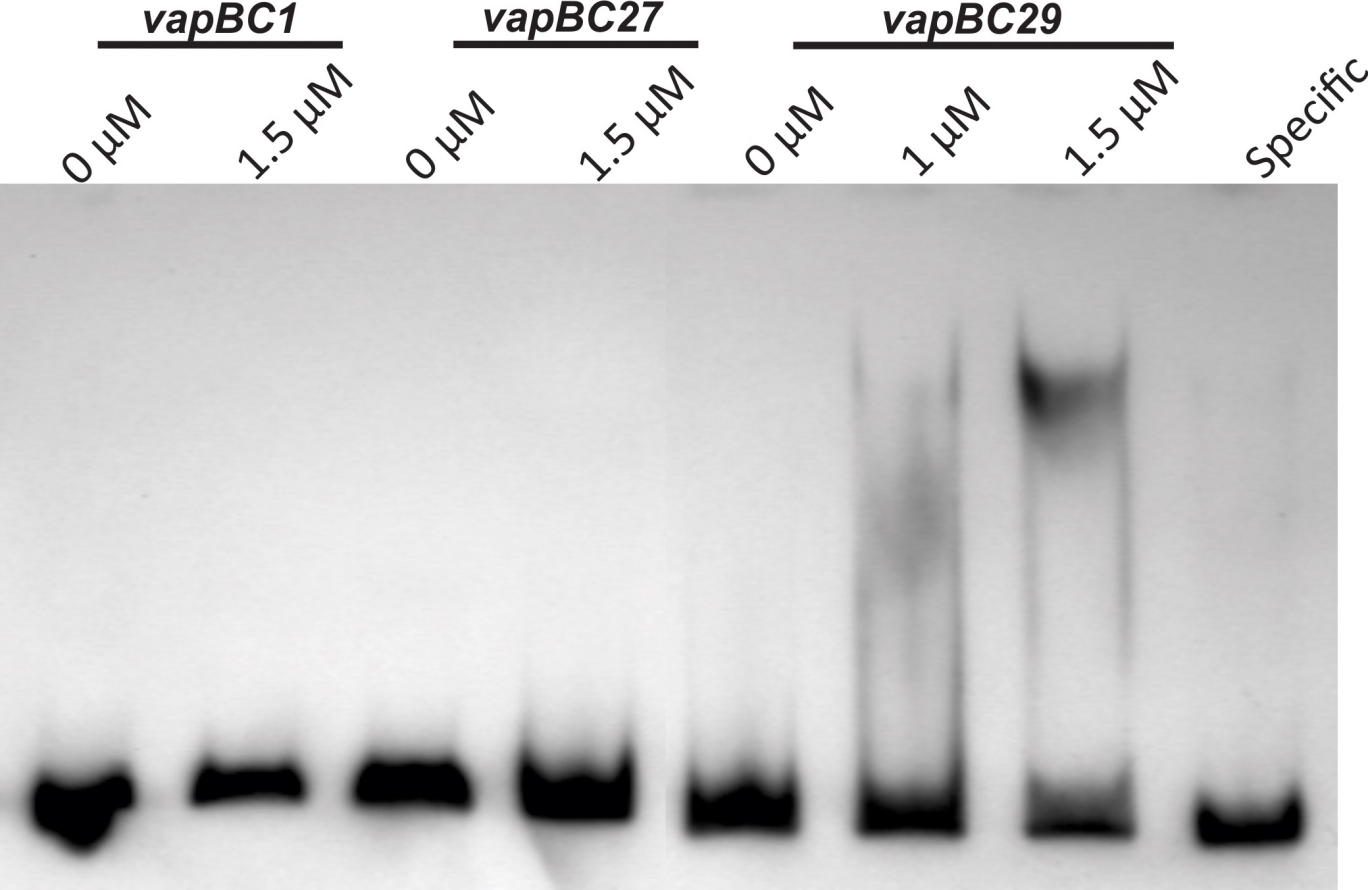
VapBC29 does not bind to *vapBC1 or vapBC27* promoters. EMSA experiments were performed to test the promiscuity of VapBC29 DNA binding. Concentrations of VapBC29 are shown above each lane. The amount of labelled DNA remained constant in each reaction (0.4 ng). Lanes 1 and 2 test binding against a 150 bp DIG-labelled region of *vapBC1* promoter DNA. Lanes 3 and 4 test binding against a 160 bp DIG-labelled region of *vapBC27* promoter DNA. Lanes 5–8 test binding against a 130 bp DIG-labelled region of *vapBC29* promoter DNA. Lane 8 specific competition from unlabelled *vapBC29* promoter DNA added in excess (200 ng).

**Table 2:** Summary of major cut sites for VapC39 on 932 RNA oligonucleotides 1–8. Cut sites determined using the improved in-house software on collected MALDI-TOF MS data.

Oligo	Major cut site target sequence	Time cut site detected
**Oligo 1**	None	N/A
**Oligo 2**	Promiscuous activity on this substrate	N/A
**Oligo 3**	AU*AU or GC*CG	15 minutes
**Oligo 4**	None	N/A
**Oligo 5**	UA*GG and UA*GG	5 minutes
**Oligo 6**	Promiscuous activity on this substrate	N/A
**Oligo 7**	UC*UA	15 minutes
**Oligo 8**	Promiscuous activity on this substrate	N/A

* denotes cleavage site.

Based on the masses of the RNA fragments analysed by MALDI-TOF MS, the mechanism of RNA cleavage by VapC from Mtb is the same as that for VapC proteins from *Pyrobaculum aerophilum* (VapC_PAE2754_, VapC_PAE0151_) and *M*. *smegmatis* (VapC_MSMEG_1284_). The phosphodiester bond is cleaved to produce a 3’OH and a 5’PO_4_, consistent with other metal-dependent enzymes [31].

### Comparison of VapC ribonuclease sequence specificity

We determined an optimal cut site of UA*GG for VapC39 and VapC27 using a combination of pentaprobe RNA oligonucleotides, mass spectrometry and polyacrylamide gel electrophoresis. Using our improved analysis method we also infer the optimal cut site for VapC29 is UA*GG. Phylogenetic analysis by Winther et al. [22] groups VapC29 with VapC37 and VapC39 in a phylogram, and as such they infer that they display the same sequence specificity. Interaction of both VapC29 and VapC37 and subsequent cleavage with tRNA-Trp^CCA^ were shown by CRAC analysis and subsequent Northern analysis. Mapping of the VapC37 cut site for tRNA-Trp^CCA^ showed cleavage at the anticodon loop at A36*A37*A38*A39, with the A37*A38 cleavage site most prominent. The cut site for VapC29 is not confirmed by Northern analysis.

We see the same shared sequence specificity with VapC29 and VapC39 but infer a target sequence of UA*GG which differs to that of the A rich sequence in the anticodon loop of tRNA-Trp^CCA^ that VapC37 targets in Winther et al [22]. It is possible that AAAA is a secondary cut site for VapC29, VapC37 and VapC39, as we see activity of VapC at UAGG sequences after a 5 minute incubation period with VapC39, but cutting at other sites after 15 minutes (Fig 3 and Table 2), with near complete degradation of the RNA oligonucleotide after 60 minutes. In comparison, tRNA-Trp^CCA^ is not completely degraded after a 120 minute assay period [22]. A confounding factor in comparing these activities is the relative stability of tRNA when compared to mRNA. For example, the half-life of mRNA in Mtb is estimated to be 9.5 minutes [32]. This disagreement immediately suggests further experiments *in vivo* in Mtb.

In our mass spectrometry data, across all pentaprobe RNA oligonucleotides tested we see many non-optimal cut sites and promiscuous activity of VapC on some of the RNA oligonucleotides (Table 2 and Figures C-F in S1 File). However, using our improved mass spectrometry data analysis pipeline which takes into account optimal and non-optimal cut sites, we see the best activity at UA*GG sequences after a five minute incubation with VapC39 and as such infer this as an optimal cut site.

### Autoregulation of *Mycobacterium tuberculosis* VapBC systems

Type II toxin-antitoxin operons are typically characterised as having tight transcriptional auto-regulation in order to ensure balanced toxin and antitoxin production. Full transcriptional repression is thought to be achieved by the toxin-antitoxin complex binding inverted repeat regulatory sequences upstream of the operon [33]. The promoter-binding capacity, and hence the potential auto-regulatory role, of VapBC1, VapBC27 and VapBC29 was investigated.

**Fig 4:**
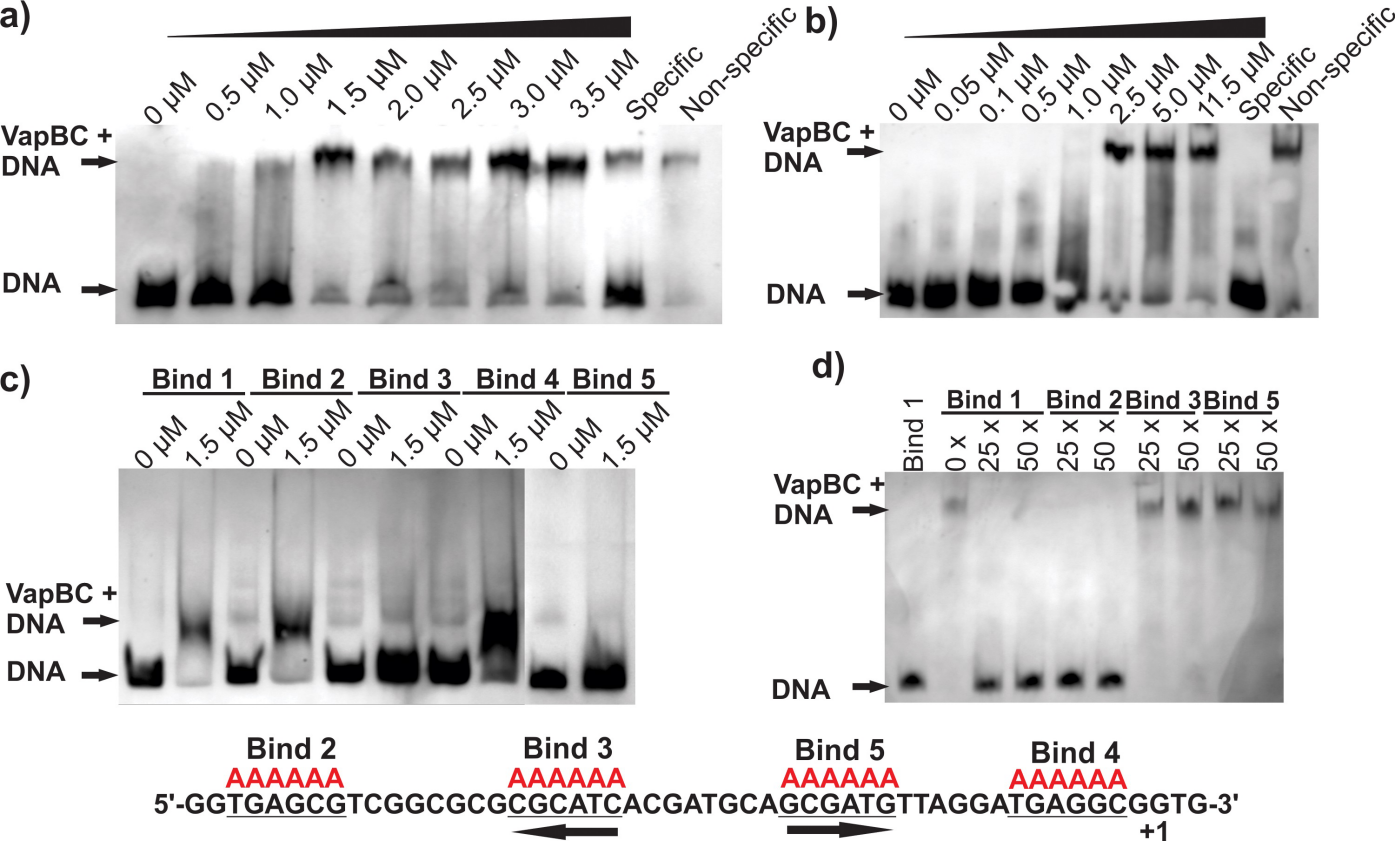
DNA-binding of VapBC29 *in vitro*. a) EMSAs demonstrate binding of the VapBC29 complex to a 130 bp *vapBC29* promoter region. Concentrations of VapBC are shown above lanes. Specific and non-specific competition were assayed using 250-fold excess (1.17 μM) of unlabelled 130 bp *vapBC29* promoter DNA (specific), or unlabelled 600 bp *Rv1494/1495 (mazEF4)* promoter DNA (non-specific). b) EMSAs demonstrate binding of the VapBC29 complex to 50 bp of DNA directly preceding the *vapBC29* transcriptional start site. Protein concentrations and competition lanes are as outlined in (a). c) EMSA experiments were performed against the 50 bp promoter region where NPIR sequences were mutated to AAAAAA (Mut.2-5); Control: non-mutated DNA (shown in black in lower panel) and Mutations 2–5 (Mut. 2–5) NPIRs underlined in the DNA sequence and mutated sequence shown in red above. d) Competition EMSA experiments were performed to test specificity of VapBC29 for NPIR sequences at Mut. 3 and Mut. 5 sites (lower panel). Each reaction contained 1.5 μM VapBC29 complex and 0.0047 μM digoxigenin-labelled control. Lane 1 represents a no protein control (labelled DNA only). Lanes 2–10 include VapBC29, labelled control DNA and an excess of unlabelled DNA (Control, Mut. 2, Mut. 3 or Mut. 5 as indicated above each lane). Unlabelled DNA was included at 25x or 50x that of the labelled control DNA.

Searches for transcriptional start sites upstream of Mtb *vapBC* operons identified putative promoters immediately upstream of each operon transcriptional start site. Electrophoretic mobility shift assays (EMSAs) were performed with purified VapBC protein (Figure A in S1 File) and corresponding *vapBC* promoter DNA. The assays demonstrated promoter-specific DNA binding activity for VapBC29, with a full shift in mobility observed at VapBC concentrations above 1.5 μM (Fig 4). In contrast, no cognate promoter binding was observed for VapBC1 nor VapBC27 (Figure G in S1 File).

The *vapBC29* promoter region tested encompassed one perfect inverted repeat (IR), however mutagenesis of this element did not abolish VapBC29 promoter binding. Analysis of the crystal structure of the FitAB TA complex from *Neisseria gonorrhoeae* bound to DNA [34] revealed that in this case specific amino acid–DNA base interactions only occur at six of the eight nucleotide positions in each IR half. As such, the putative *vapBC29* promoter was re-analysed for the presence of ‘non-perfect inverted repeats’ (NPIRs) as potential VapBC binding sites. Eleven NPIRs were found to be present that agreed with the following parameters: at least five bp in length, no more than two base mismatches between halves and a spacer of at least five bp between halves (Table C in S1 File).

In order to elucidate which NPIR was predominantly involved in VapBC29 promoter binding, smaller DNA oligos were designed to span the original VapBC29 promoter region. EMSAs with these smaller oligos revealed VapBC29 binding to be specific to the 50 bp of DNA directly preceding the VapBC29 transcriptional start site (Fig 4B). This 50 bp oligo encompassed four of the 11 identified NPIRs. In order to ascertain the importance of each NPIR in the DNA-binding interaction, variants of the oligo were created with mutations introduced at each of the candidate NPIRs (Fig 4C). EMSAs with these DNA variants implicated a six bp NPIR sequence ^5’^CATCAC–N_6_-GCGATG^3^’ that is only found once in the Mtb genome and is essential for the maintenance of VapBC29 promoter binding. When either repeat (CATCAC or GCGATG) was mutated no shift was observed in the DNA when incubated with VapC29 (Mutations 3 and 5 Fig 4C). Competition experiments with unlabelled competitor DNA show specificity of VapBC29 for the NPIR sequence ^5’^CATCAC–N_6_-GCGATG^3^’ (Mutations 3 and 5 in Fig 4D). One half of this NPIR overlaps a previously annotated -10 mycobacterial promoter element, GATGTT (‘T26’), corroborating the prediction that VapBC binding at this site would interfere with transcription.

While VapB27 is identified as a member of the AbrB/MazE superfamily (COG2002), VapB1 and VapB29 have no conserved domains consistent with currently identified families. Slightly differing stoichiometry between the three systems (Table 1 and Figure A in S1 File) may reflect the ability of the VapBC complex to bindDNA. Additionally, and unlike for VapBC29, no similarity was found between the VapBC1 and VapBC27–10 and -35 sequence regions and those reportedly seen in other mycobacterial promoters. It may be that the VapBC1 and VapBC27 systems require an additional interaction to take place for DNA binding to occur, or that a completely different mechanism of auto-regulation is implemented. Individualised transcriptional regulation mechanisms among the VapBC systems of Mtb may add an extra layer of complexity to the regulation of the VapBC TA system network, perhaps helping to explain the persistence of such an expanded number of these operons in the genome.

### Regulation of the VapBC29 operon is specific

Previous studies have revealed evidence of some ‘cross-talk’ among non-cognate type II toxin-antitoxin system components in Mtb, both in terms of formation of hetero-familial TA complexes, and in the binding of TA complexes to non-cognate promoter regions for regulatory purposes [35–37]. For example, the evidence of interaction between MazF toxins and VapB antitoxins [38], and among members of the RelBE system [39]. The potential formation of TA complexes between non-cognate toxins and antitoxins adds an additional layer of complexity to the intricately orchestrated Mtb TA network. Inter-system relationships may have evolved to allow fine-tuning of the delicate toxin:antitoxin ratio in the cell that is required for an accurate response to environmental cues. On the other hand, it has also been suggested that involvement of a certain antitoxin in the functioning of multiple TA systems may in fact lead to redundancy of its cognate toxin and in turn the decay of this system [40].

VapBC29 binding promiscuity was investigated by conducting EMSAs with the promoter regions of *vapBC1* and *vapBC27* (Fig 5). No binding of VapBC29 was observed for *vapBC1* and *vapBC27* promoter sequences (Fig 5). Addition of excess unlabelled *vapBC29* promoter DNA abolishes the shift, indicating specificity for this promoter region (Fig 5, Specific Lane). In addition, the non-specific control used for characterising VapBC29 interactions with sequences in the *vapBC2*9 promoter was DNA from the *mazEF4* (*rv1494/5*) promoter (Fig 4A and 4B). Binding of VapBC29 to its cognate promoter DNA was still observed in the presence of *mazEF4* promoter DNA, suggesting that VapBC29 does not bind to the *mazEF4* promoter sequence also. Overall, this suggests that the binding of the VapBC29 complex is restricted to its cognate promoter.

### Differential growth inhibition in *Mycobacterium smegmatis*

Studies conducted on a number of the Mtb VapBC systems have revealed that some, but not all VapC proteins inhibit growth when overexpressed in mycobacterial species [23, 35, 41]. VapC proteins VapC1, VapC27 and VapC29 were conditionally expressed in *M*. *smegmatis* using the tetracycline inducible pMIND plasmid [42]. Growth was monitored in liquid media by Optical Density (OD) measurements and on solid media by CFU measurements. Toxicity was classified as either bacteriostatic or bactericidal depending on whether or not *M*. *smegmatis* growth could be rescued upon repression of VapC expression.

**Fig 6:**
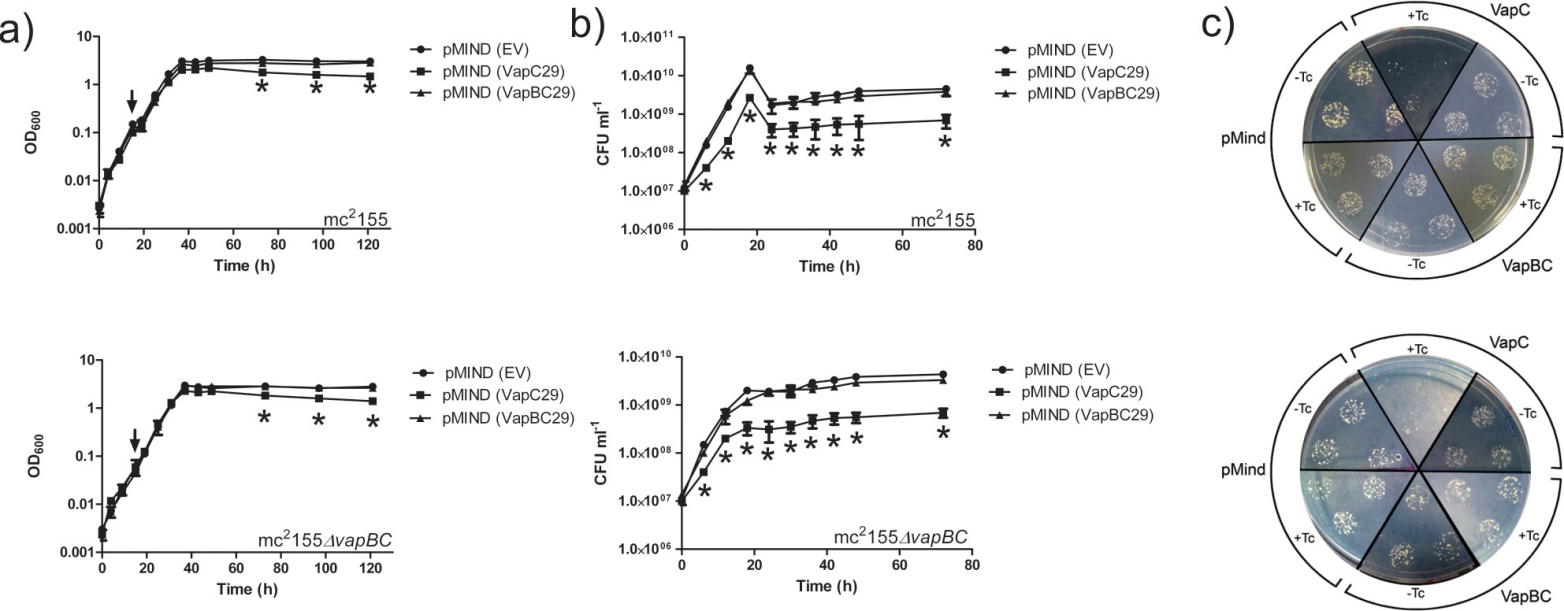
The effect of VapC29 overexpression on the growth and viability of *M*. *smegmatis* mc^2^155 and Δ*vapBC* strains. The effect of conditionally expressing VapC29, VapBC29 or empty vector (EV) on growth (a) and cell viability (b) was examined in a wild-type and *vapBC* knockout strain of *M*. *smegmatis* mc^2^155. Protein expression was induced during early exponential growth by the addition of 20 ng.ml^-1^ tetracycline (indicated in (a) by a black arrow). CFU viability measurements were taken in the 96 hour period following. Results shown indicate the mean ± SD of three technical replicates of each of three biological replicates. Significant (p < 0.05) points (as determined by one-way ANOVA) are shown by an *. c) A mosaic of agar plates showing colony growth of the *M*. *smegmatis* mc^2^155 strain and the *M*. *smegmatis* mc^2^155 Δ*vapBC* strain containing empty pMind, pMind_VapC29 or pMind_VapBC29 in the presence (+Tc) and absence (-Tc) of tetracycline. Each segment includes three technical replicates of *M*. *smegmatis* culture growth at 48 hours post-induction diluted 10,000 fold.

Expression of VapC29 inhibited growth in both *M*. *smegmatis* mc^2^155 (wild-type) and *ΔvapBC* strains (Fig 6) with the degree of VapC29 toxicity more apparent when comparing CFUs (Fig 6B). At 96 hours post-induction, the wild-type strain expressing VapC29 exhibited an 87-fold reduction in cell viability compared to cells carrying the empty plasmid, and an 86-fold reduction compared to the strain expressing the VapBC29 complex. Similarly, induction of VapC29 in the *ΔvapBC* strain led to a 86-fold and 82-fold reduction in cell viability when compared to strains expressing empty plasmid and VapBC29 respectively (Fig 6B). In contrast, strains expressing VapC29 in complex with VapB29 showed no effect on growth, indicating abrogation of VapC toxicity through binding to VapB (Fig 6). Cells containing the VapC29-pMIND plasmid formed very small ‘pin-prick’ colonies when grown on agar plates in the presence of tetracycline (Fig 6C). This morphology was not seen when a VapC29 expression culture was streaked on a plates lacking tetracycline, indicative of a bacteriostatic rather than bactericidal mechanism of growth inhibition by VapC29.

Neither wild-type nor *ΔvapBC* strains expressing VapC1, VapC27, VapBC1 and VapBC27 exhibited any difference in growth or colony morphology compared to strains with the empty plasmid (Figures H and I in S1 File).

A toxicity screen of putative Mtb VapC proteins conducted by Ramage et al (2009) [35] reported all three of these VapC proteins to lack toxic activity *in vivo* in *M*. *smegmatis*. The discrepancy for VapC29 between our study and theirs is most likely due to the use of alternate inducible expression and toxicity analysis protocols. Here we assess growth in both liquid and solid media, and use a bioavailable inducer (tetracycline), so the toxicity of VapC29 is possibly more apparent. Winther et al (2016) also conducted toxicity screens of Mtb VapC proteins in *M*. *smegmatis* using the pMEND-HTF plasmid that also has a tetracycline inducible promoter but differs in that it introduces a hexa-His, TEV cleavage site and 3 x FLAG tags at the C-terminus of the protein (HTF-tag) [22]. As in the experiment presented here, they show that VapC29 strongly inhibits cell growth, whereas VapC1 and VapC 27 have no effect on growth [22].

## Conclusions

The role of VapBC systems in Mtb has been the subject of great interest over recent years, due to their expanded number in the genome of this pathogenic organism and links with the regulation of cell growth in response to environmental stress. VapC1, 19, 27, 29, 39 are all magnesium-dependent ribonucleases with identical sequence specificity, targeting UA*GG sequences. In all cases, the VapB and VapC products of each operon interact to form a VapBC complex which lacks ribonuclease activity. VapC29 displayed toxicity *in vivo* when conditionally expressed in *M*. *smegmatis* while VapC1 and VapC27 did not, despite all three exhibiting identical sequence specificity *in vitro*. VapBC29 is auto-regulatory, as shown by EMSA experiments with *vapBC29* promoter DNA. Interactions with promoter DNA display typical TA systems characteristics; whereby the VapBC complex bound specifically to the inverted repeat ^5’^CATCAC–N_6_-GCGATG^3^ in the *vapBC29* promoter. The fact that these five Mtb VapC toxins tested thus far appear to target the same primary mRNA sequence is intriguing. Maintaining a suite of genes with apparent redundancy in activity would be seemingly costly to the organism in terms of energy and maintenance. This raises the possibility that additional layers of specificity and control come in to play which may segregate Mtb VapC into sub-groups, each of which are activated under different conditions. This is seen in Gupta et al (2017) where TA activation is stress specific, with different TA loci being activated in response to a particular stress [43]. In addition, a TrASH analysis for the genetic requirements for fast and slow growth in *Mycobacterium bovis* BCG showed that VapB27 is required for the slow to fast growth rate switch [44] whereas the other VapBC systems investigated in this study were not required for the switch. Recognition of unique secondary structural motifs of RNA substrates along with individualised auto-regulatory mechanisms and differences in activity may lead to such segregation of TA systems. The next challenge will be to further investigate the molecular processes signalling the full activation of these systems, and identify their cellular targets.

## Supporting information

S1 File Supplementary figures. (DOCX)
