# Supplementary material for: VapC proteins from Mycobacterium tuberculosis share ribonuclease sequence specificity but differ in regulation and toxicity
Source: PLoS One. 2018 Aug 31;13(8):e0203412. doi: 10.1371/journal.pone.0203412 (PMC6118392; doi:10.1371/journal.pone.0203412)
Supplement: S1 File — (DOCX) [file pone.0203412.s001.docx]

Supplementary Tables and Figures:

**Table A.** Primers and oligonucleotides used in this study.

| Primer Name | **Sequence** |
| --- | --- |
| Rv0064 F | GTTACCATGGCTACCATTCAAGTTCGGGA |
| Rv0065 R | GGCAAGCTTCCGAACGAGTTTGATTTCG |
| Rv0616 F | TAATCCATGGTGCGCACTACCATCGA |
| Rv0617 R | TAAAGCTTGGTGGTCGTTGGAATGAG |
| Rv0599 F | AGAACCATGGCGGCTGTTGTGGATGC |
| Rv0598 R | TTAAAGCTTCTCGAGCATCGCGTGTGG |
| Rv2530A F | ATTACCATGGCCACCACGTTGCAGATTGAT |
| Rv2530c R | TATAAGCTTCAACACCTCGACGAGGCC |
| Rv2548 F | ATTGCCATGGCTGGTGATGTCATGCGGA |
| Rv2549 R | ATTGCATATGGTACGGCGGCTGCAGATC |
| Rv0616/0617 F | AAATTTGGATCCGGAGGAATAATGGTGCGCACTACCATC |
| Rv0617 F | AAATTTGGATCCGGAGGAATAATGGTGACGGTGCTGCT |
| Rv0616/0617 R | AAATTTACTAGTTCATTGGTGGTCGTTGGAATGAGTACC |
| Rv0064/0065 F | AAATTTGGATCCGGAGGAATAATGGCTACCATTCAAGT |
| Rv0065 F | AAATTTGGATCCGGAGGAATAATGGTGGATGAATGTGTA |
| Rv0064/0065 R | AAATTTACTAGTTCACCGAACGAGTTTGATTTCGCACG |
| Rv0598/0599 F | AAATTTGGATCCGGAGGAATAATGAAGGCTGTTGTGGATGC |
| Rv0598 F | AAATTTGGATCCGGAGGAATAATGGTGAAACCGCCGCTGGCAGT |
| Rv0598/0599 R | AAATTTACTAGTTCACGCGGCGACAACGAC |
| Rv0065promoter F | CTGGCCGATCTGCGCGCGGT |
| Rv0065promoter dstr F | GTGGATAAGCCGGCATTCTTAGCCG |
| Rv0617promoter F | CCGCCACCCAACACGGCTCGT |
| Rv1494promoter F | CGAGATCTCCGAAGCGCTGGAGAAG |
| Rv0065promoter R | AGCGGAGTTCACCGGCTAAGAATGCCG |
| Rv0065promoter dstr R | TCGATGAGCTTGGTGCGCATATACG |
| Rv0617promoter R | GCCTCATCCTAACATCGCTGCATCGTGATGC |
| Rv1494promoter R | CGCGGGCCTCTCGCTAATCCAGCGTG |
| Rv0599promoter F | ACGTCATCAGATACAAAGGACCCGA |
| Rv0599promoter R | CATGGGAATACGATACCAATGGCTTCC |
| Oligo 1F | CCGCCACCCAACACGGCTCGTGCGCTGTCTCGGCCGGCTC |
| Oligo 2F | CTCGTGCGCTGTCTCGGCCGGCTCGTCTGCCGCACGGCCA |
| Oligo 3F | GCTCGTCTGCCGCACGGCCAGCATGATCAGTCCCGTTGGA |
| Oligo 4F | CACGGCCAGCATGATCAGTCCCGTTGGAATACCGGTGAGCGTCGGCGCGC |
| Oligo 5F | GGTGAGCGTCGGCGCGCGCATCACGATGCAGCGATGTTAGGATGAGGCGG |
| Oligo 5 Bind 2F | GGAAAAAATCGGCGCGCGCATCACGATGCAGCGATGTTAGGATGAGGCGG |
| Oligo 5 Bind 3F | GGTGAGCGTCGGCGCGAAAAAAACGATGCAGCGATGTTAGGATGAGGCGG |
| Oligo 5 Bind 4F | GGTGAGCGTCGGCGCGCGCATCACGATGCAGCGATGTTAGGAAAAAAAAG |
| Oligo5 Bind 5F | GGTGAGCGTCGGCGCGCGCATCACGATGCAAAAAAATTAGGATGAGGCGG |
| Oligo 1R | GAGCCGGCCGAGACAGCGCACGAGCCGTGTTGGGTGGCGG |
| Oligo 2R | TGGCCGTGCGGCAGACGAGCCGGCCGAGACAGCGCACGAG |
| Oligo 3R | TCCAACGGGACTGATCATGCTGGCCGTGCGGCAGACGAGC |
| Oligo 4R | GCGCCGACGCTCACCGGTATTCCAACGGGACTGATCATGCTGGCCGTG |
| Oligo 5R | CCGCCTCATCCTAACATCGCTGCATCGTGATGCGCGCGCCGACGCTCACC |
| Oligo 5 Bind 2R | CCGCCTCATCCTAACATCGCTGCATCGTGATGCGCGCGCCGATTTTTTCC |
| Oligo 5 Bind 3R | CCGCCTCATCCTAACATCGCTGCATCGTTTTTTTCGCGCCGACGCTCACC |
| Oligo 5 Bind 4R | CTTTTTTTTCCTAACATCGCTGCATCGTGATGCGCGCGCCGACGCTCACC |
| Oligo 5 Bind 5R | CCGCCTCATCCTAATTTTTTTGCATCGTGATGCGCGCGCCGACGCTCACC |

**Table B. Summary of small scale expression tests and His-tag binding for 47 Mtb VapBC complexes.** VapBC operons in bold type indicate soluble VapC protein. * indicates VapB fused to VapC, ** indicates the operon has been previously cloned in our laboratory. Soluble and insoluble expression of VapB and VapC proteins with molecular weights described.

| **Operon** | **VapB** | | | | **VapC** | | | |
| --- | --- | --- | --- | --- | --- | --- | --- | --- |
|  |  | **Expected size (kDa)** | **Insoluble Expression** | **Soluble Expression** |  | **Expected size (kDa)** | **Insoluble Expression** | **Soluble Expression** |
| Rv0064/0065 | Rv0064 | 8.7 | ✓ | ✓ | Rv0065 | 15.8 | x | **✓** |
| Rv0229c* | - | - | - | - | Rv0229c | 26.8 | x | x |
| Rv0239/0240 | Rv0239 | 11.5 | x | ✓ | Rv0240 | 17.9 | x | **✓** |
| Rv0277a/0277c | Rv0277a | 6.0 | x | x | Rv0277c | 17.4 | x | x |
| Rv0300/0301 | Rv0300 | 8.1 | ✓ | ✓ | Rv0301 | 17.2 | x | **✓** |
| Rv0549/0550 | Rv0550 | 9.5 | ✓ | x | Rv0549c | 17.7 | ✓ | x |
| Rv0582/0581 | Rv0581 | 7.6 | x | x | Rv0582 | 15.9 | x | x |
| Rv0595/0596c | Rv0596c | 9.7 | x | x | Rv0595c | 15.6 | x | x |
| Rv0598c/0599 | Rv0599 | 8.2 | x | ✓ | Rv0598c | 17.2 | x | **✓** |
| Rv0609/0608 | Rv0608 | 8.8 | x | x | Rv0609 | 16 | x | x |
| Rv0617/0616 | Rv0616 | 8.2 | ✓ | ✓ | Rv0617 | 15.5 | x | **✓** |
| Rv0623/0624** | Rv0623 | 9.1 | x | x | Rv0624 | 17.3 | x | x |
| Rv0627/0626 | Rv0626 | 9.5 | x | x | Rv0627 | 15.9 | x | x |
| Rv0656/0657 | Rv0657 | 11.1 | ✓ | x | Rv0656c | 16.9 | x | x |
| Rv0661c/0662c | Rv6062c | 14 | x | x | Rv0661 | 16.8 | x | x |
| Rv0665/0664 | Rv0064 | 9.2 | x | x | Rv0665 | 13.1 | x | **✓** |
| Rv0749/0748 | Rv0748 | 9 | x | x | Rv0749 | 17.4 | x | x |
| Rv0960/0959a | Rv0959a | 7.9 | x | x | Rv0960 | 15.4 | x | x |
| Rv1114/1113 | Rv1113 | 6.9 | ✓ | ✓ | Rv1114 | 15 | ✓ | **✓** |
| Rv1242/1241 | Rv1241 | 9.8 | x | x | Rv1242 | 17.4 | x | x |
| Rv1397c/1398c | Rv1398c | 9.4 | x | x | Rv1397c | 16.5 | x | **✓** |
| Rv1561/1560 | Rv1560 | 8.3 | ✓ | x | Rv1561 | 16.2 | x | x |
| Rv1720/1721c | Rv1721c | 7.9 | x | x | Rv1720 | 15.4 | x | x |
| Rv1741/1740 | Rv1740 | 7.6 | x | x | Rv1741 | 10.4 | ✓ | x |
| Rv1838c/1839c | Rv1839c | 10.4 | x | ✓ | Rv1838c | 16.2 | x | x |
| Rv1953/1952 | Rv1952 | 7.8 | x | x | Rv1953 | 13.1 | x | x |
| Rv1962c/1962a | Rv1962a | 9.9 | ✓ | ✓ | Rv1962c | 16 | x | **✓** |
| Rv1982c/1982a | Rv1982a | 9.6 | ✓ | x | Rv1982c | 17.1 | ✓ | x |
| Rv2010/2009 | Rv2009 | 9.0 | ✓ | ✓ | Rv2010 | 16.3 | ✓ | x |
| Rv2103c/2104c | Rv2104c | 9.3 | ✓ | x | Rv2103c | 18.1 | x | **✓** |
| Rv2494/2493 | Rv2493 | 8.0 | ✓ | x | Rv2494 | 16.9 | x | **✓** |
| Rv2527/2526 | Rv2526 | 8.2 | ✓ | ✓ | Rv2527 | 16.7 | ✓ | **✓** |
| Rv2530c/2530a | Rv2530a | 7.9 | x | x | Rv2530 | 16.3 | ✓ | **✓** |
| Rv2546/2545 | Rv2545 | 10.3 | ✓ | x | Rv2546 | 16.6 | x | x |
| Rv2548/2547 | Rv2547 | 9.9 | ✓ | ✓ | Rv2548 | 17.4 | ✓ | **✓** |
| Rv2549c/2550c | Rv2550c | 9.1 | x | x | Rv2549c | 16.1 | ✓ | x |
| Rv2596/2595 | Rv2595 | 9.2 | x | x | Rv2596 | 15.9 | x | **✓** |
| Rv2602/2601 | Rv2601 | 10.4 | x | x | Rv2602 | 17.6 | x | x |
| Rv2757/2758c | Rv2758c | 9.4 | x | x | Rv2757 | 18.2 | x | x |
| Rv2759c/2760c | Rv2760c | 10.1 | x | x | Rv2759c | 15.9 | x | x |
| Rv2829c/2830c | Rv2830c | 7.5 | x | x | Rv2829c | 16.1 | x | x |
| Rv2863/2862a | Rv2862a | 7.4 | x | x | Rv2863 | 15.8 | x | x |
| Rv2872/2871 | Rv2871 | 9.1 | x | x | Rv2872 | 18.1 | x | x |
| Rv3320c/3321 | Rv3321 | 8.8 | x | x | Rv3320c | 18.2 | x | x |
| Rv3384c/3385c | Rv3385c | 11.0 | x | ✓ | Rv3384c | 15.8 | x | **✓** |
| Rv3408/3407 | Rv3407 | 10.9 | ✓ | ✓ | Rv3408 | 16.2 | ✓ | **✓** |
| Rv3697c/3697a | Rv3697a | 8.4 | x | x | Rv3697c | 17.8 | x | x |

**Table C. Properties of NPIRs in the 130 bp *vapBC29* promoter region**

| IR Code  (5’-3’) | Genomic position from TSP^*^ | Mismatches (bp) | IR Length (bp) | Spacer Length (bp) | Full IR covered in Oligo |
| --- | --- | --- | --- | --- | --- |
| A | -98, -118 | 2 | 6 | 14 | 1 |
| B1^**^ | -103, -114 | 1 | 5 | 6 | 1 |
| B2** | -77, -103 | 1 | 5 | 21 | 2 |
| C | -60, -80 | 2 | 6 | 14 | 2 |
| D | -36, -75 | 2 | 6 | 33 | 3 |
| E | -56, -73 | 1 | 5 | 12 | 3,4 |
| F | -45, -64 | 3 | 8 | 11 | 4 |
| G | -48, -58 | 1 | 5 | 5 | 4 |
| H | -30, -42 | 1 | 6 | 6 | 5 |
| I | -27, -1 | 1 | 7 | 19 | 5 |
| J | -14, -26 | 1 | 6 | 6 | 5 |
| *Genomic positions were measured by counting from TSP (GTG) to the 3’-most base of each IR half  **B1 and B2 share one half of their IR sequence | | | | | |


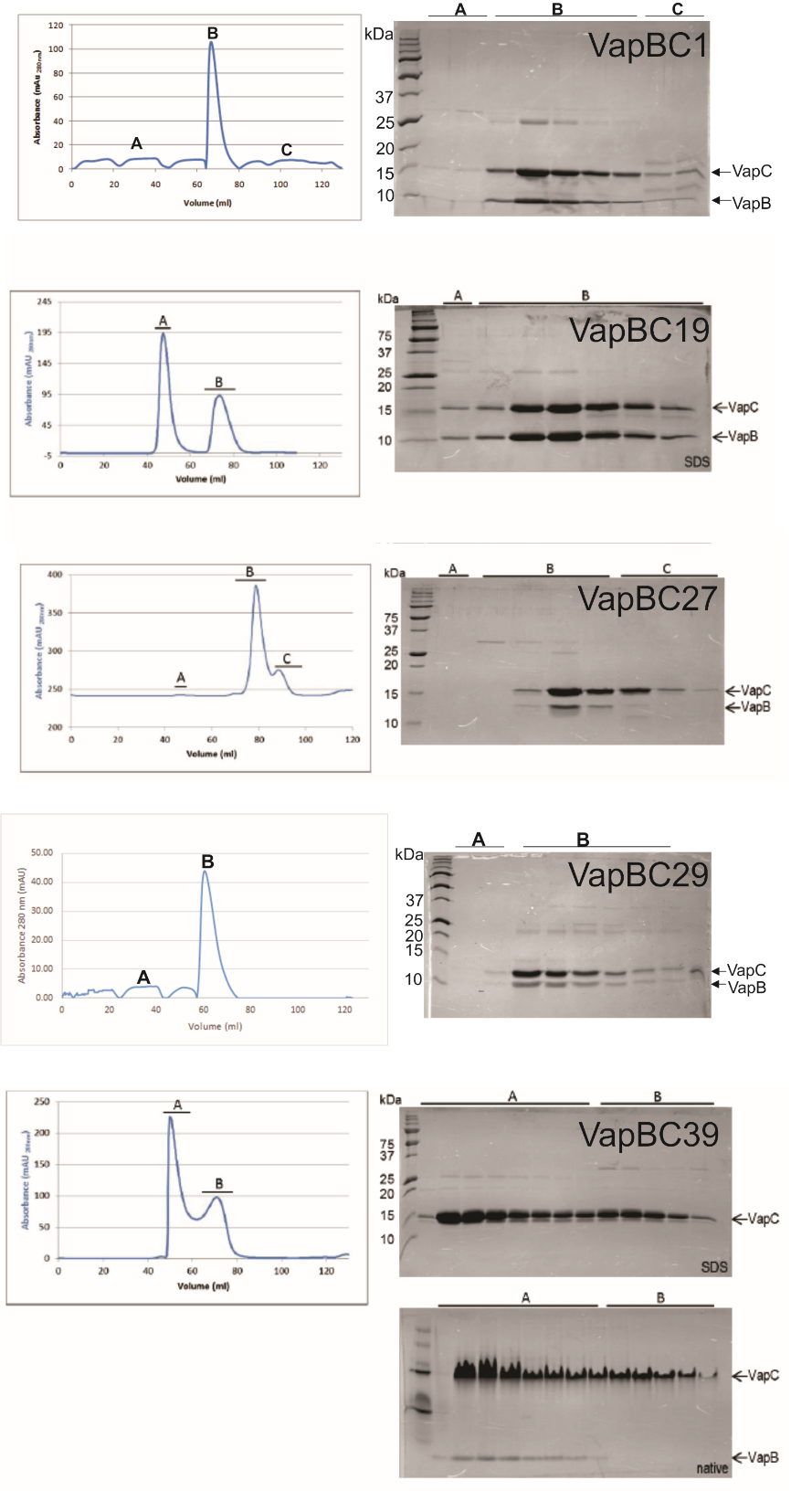


**Figure A. SEC Purification of VapBC1, VapBC27 and VapBC29 TA complexes**. S200 16/60 gel filtration column purification of nickel purified VapBC complexes. Molecular weight markers on the side of each SDS-PAGE gel, and positions of VapC and VapB proteins noted.


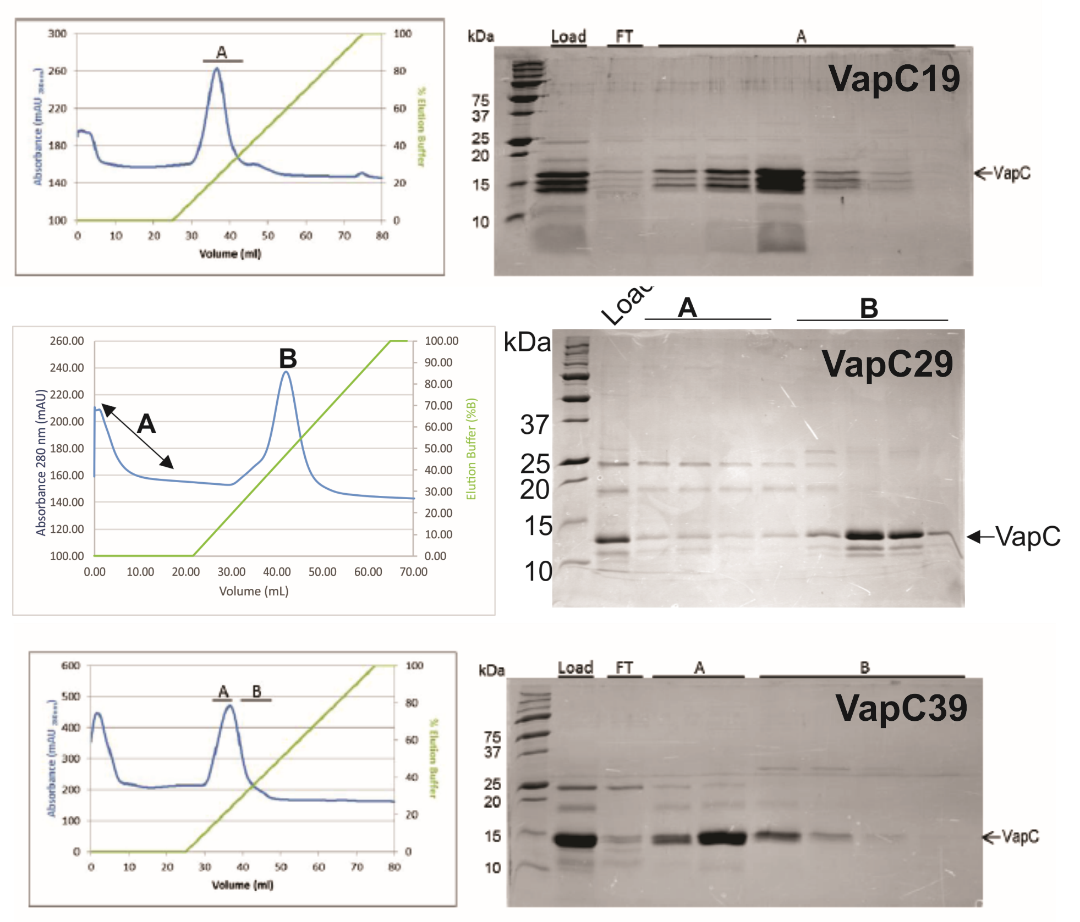


**Figure B. Anion Exchange Chromatography and SDS-PAGE analysis of VapC proteins**. Purification of trypsin digested VapBC complexes (Load) using anion exchange chromatography to remove trypsin. Molecular weight markers on the side of each SDS-PAGE gel, and positions of VapC proteins noted.


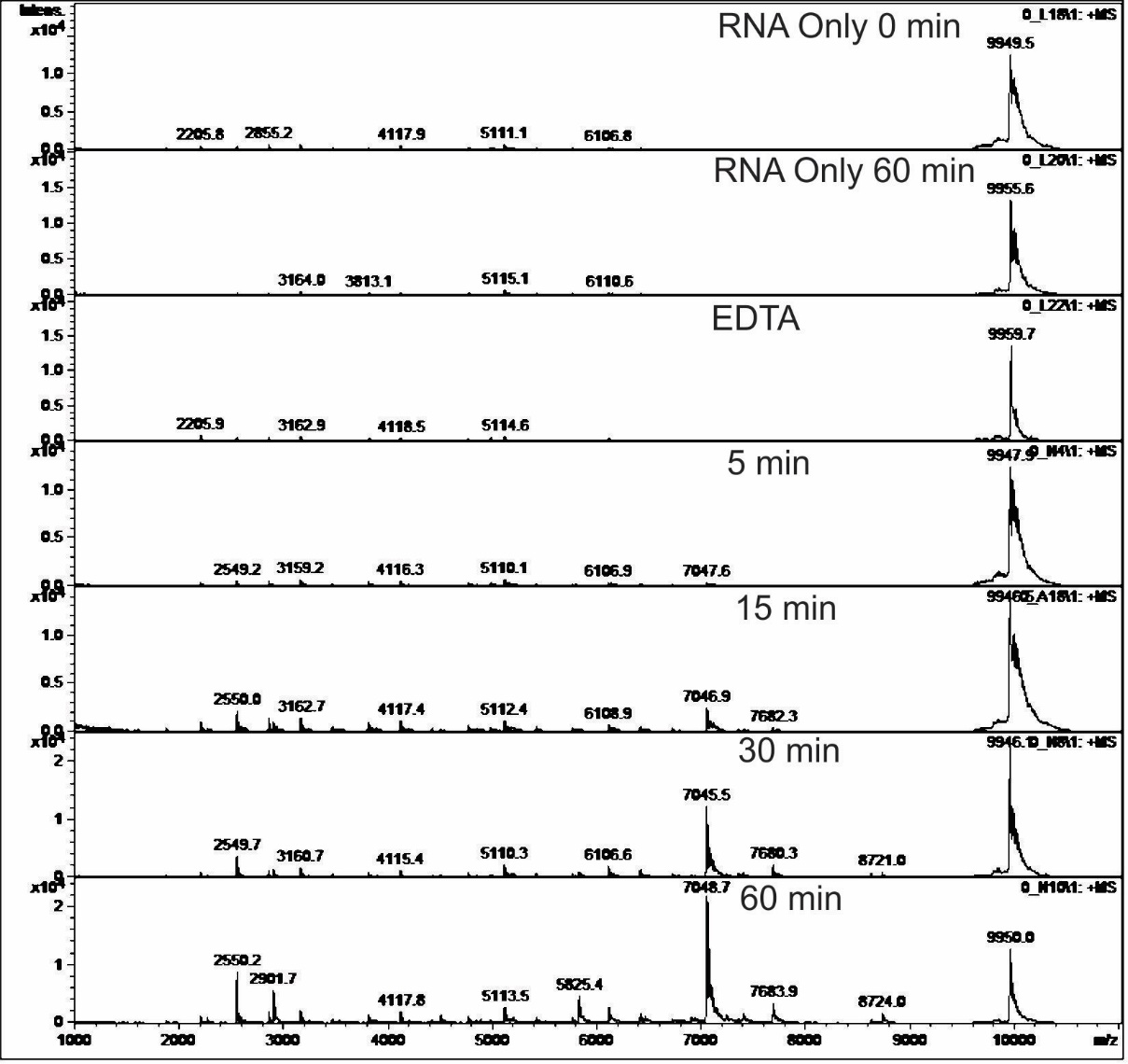


**Figure C. VapC39 ribonuclease assay MALDI-TOF MS spectra RNA oligonucleotide 6.** MALDI-TOF MS analysis of Pentaprobe RNA Oligo 6 Ribonuclease assay for VapC39


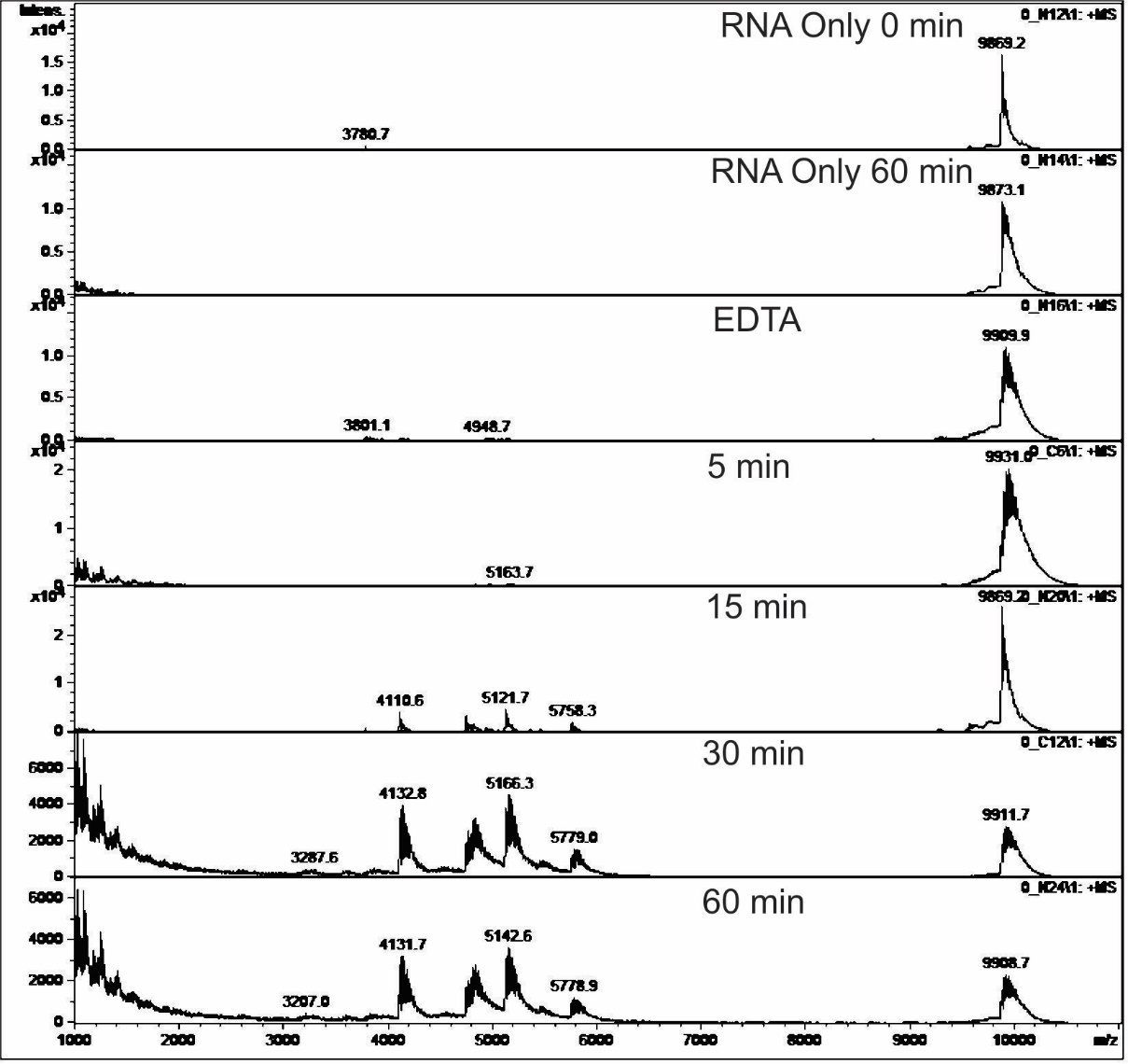


**Figure D**. **VapC39 ribonuclease assay MALDI-TOF MS spectra RNA oligonucleotide 7.** MALDI-TOF MS analysis of Pentaprobe RNA Oligo 7 Ribonuclease assay for VapC39


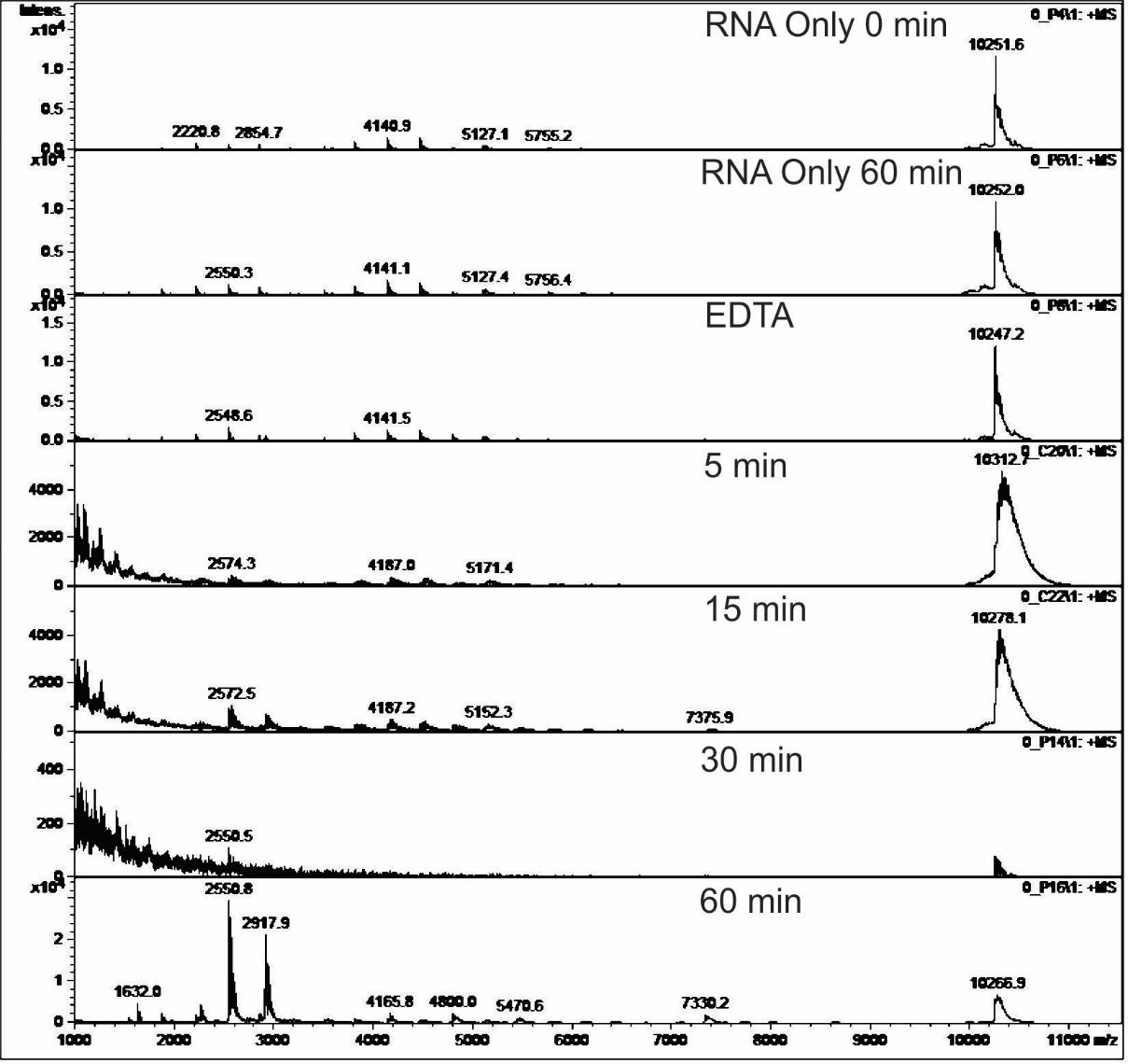


**Figure E**. **VapC39 ribonuclease assay MALDI-TOF MS spectra RNA oligonucleotide 8.** MALDI-TOF MS analysis of Pentaprobe RNA Oligo 8 Ribonuclease assay for VapC39

**Figure F**. **VapC1 and VapC29 ribonuclease assay MALDI-TOF MS spectra.** MALDI-TOF MS analysis of Pentaprobe RNA Oligo 5 Ribonuclease assay for VapC1 (labelled Rv0065) and VapC29 (Rv0617) is identical to the spectra obtained for VapC39 (Fig 3).

**
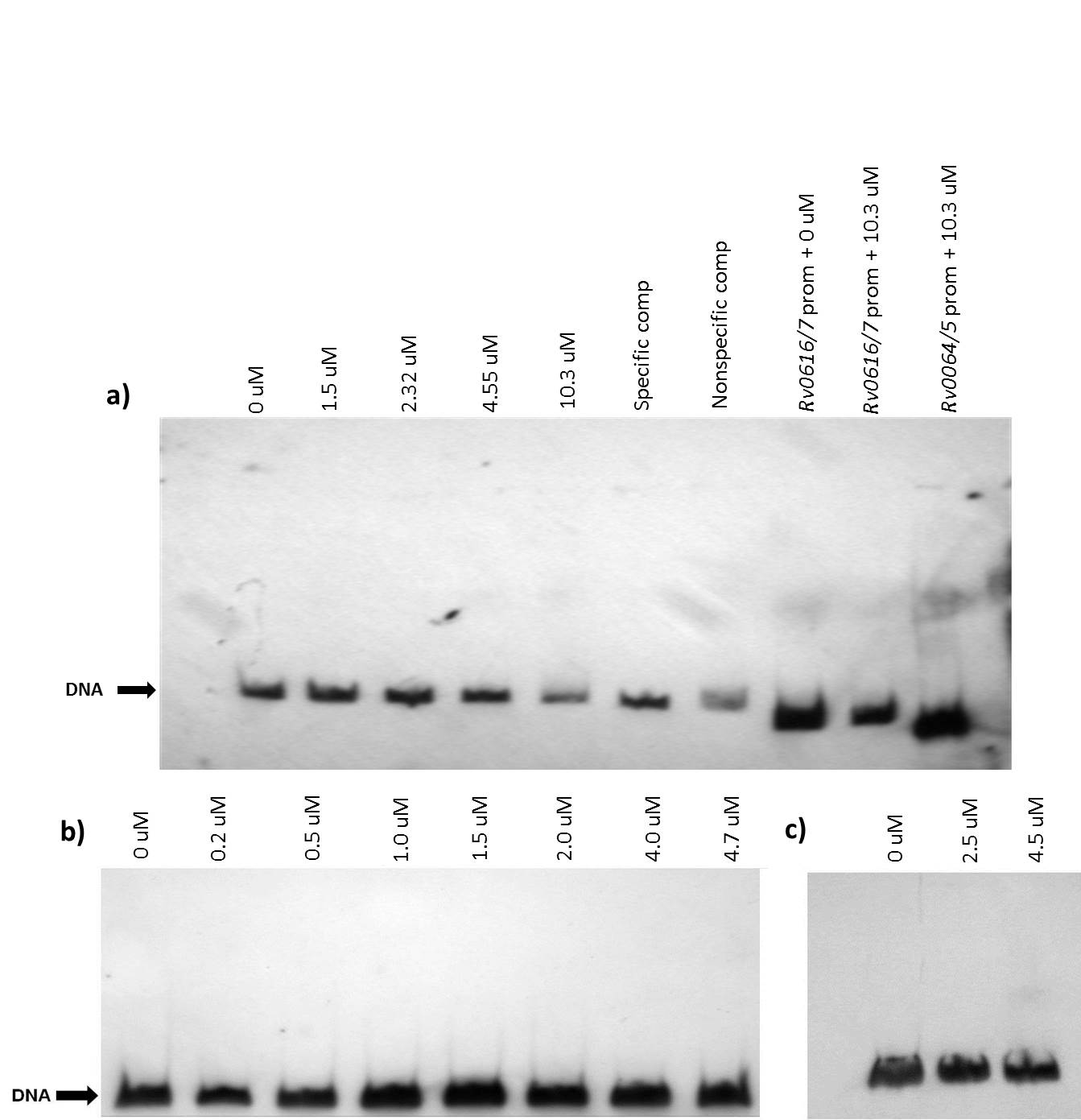
**

**Figure G. VapBC27 and VapBC1 bind to neither the *vapBC27, vapBC29* or *vapBC1* promoter regions.** a) VapBC27 EMSA experiments were performed with a 160 bp DIG-labelled region of *vapBC27* promoter DNA (lanes 1 – 7). EMSA experiments were repeated testing VapBC27 against either a 130 bp DIG-labelled region of *vapBC29 ­*promoter (lanes 8,9) or a 101 bp DIG-labelled region of *vapBC1 ­*promoter DNA (lane 10). VapBC27 -DNA binding was absent in all cases as shown by an absence of shift on the gel. b) VapBC1 EMSA experiments were performed with a 101 bp DIG-labelled region of *vapBC1* promoter DNA. c) VapBC1 EMSA experiments were performed with a 150 bp DIG-labelled region of *vapBC1*promoter DNA. a) – c) Concentrations of VapBC27 are shown above each lane. The amount of DNA (0.4 ng) remained constant in each reaction.


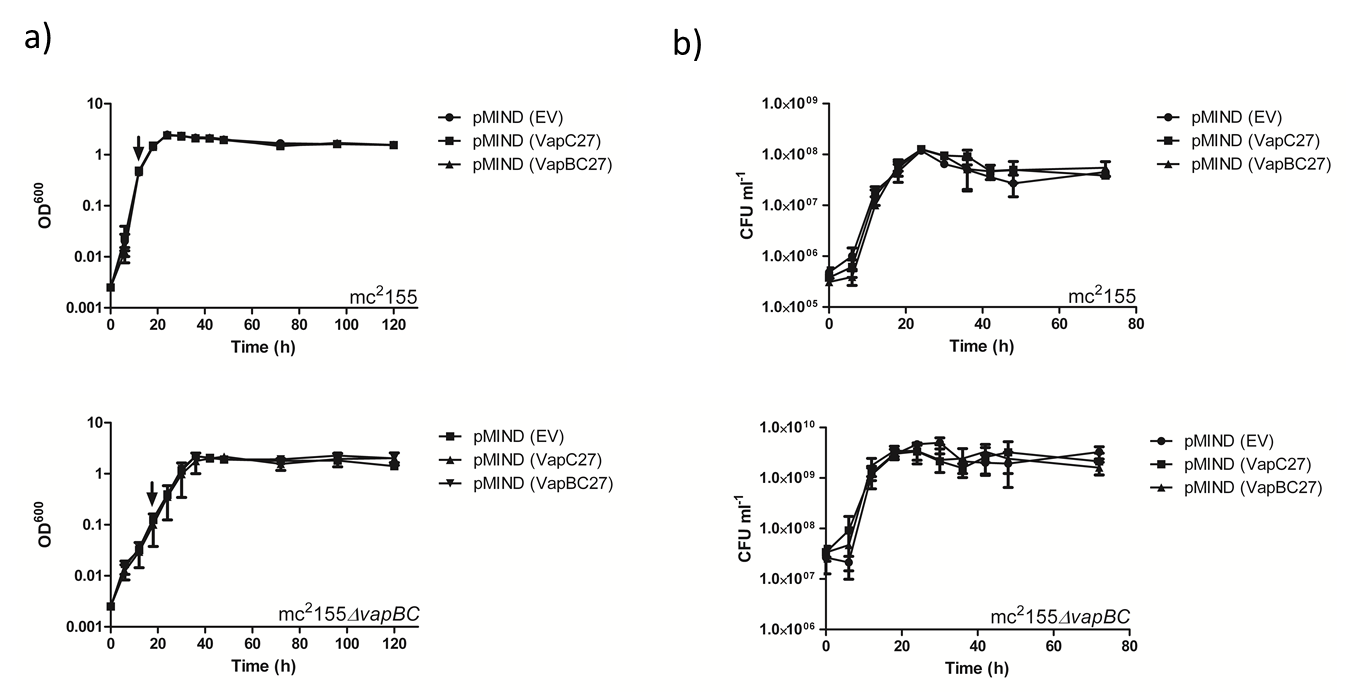


**Figure H. The effect of VapC27 overexpression on the growth and viability of *M. smegmatis* mc^2^155 and ∆*vapBC* strains.** The effect of conditionally expressing VapC27, VapBC27 or empty vector (EV) on growth (a) and cell viability (b) was examined in a wild-type and *vapBC* knockout strain of *M. smegmatis* mc^2^155. Protein expression was induced during early exponential growth (OD_600_ of 0.1-0.2) by the addition of 20 ng.ml^-1^ tetracycline (indicated in a) by black arrow). CFU viability measurements were taken in the 96 hour period following. Data were plotted in Prism V7. Graphs shown are from data acquired from one independent experiment representative of three biological replicates. Results shown indicate the mean ± SD of three technical replicates of each of three biological replicates.


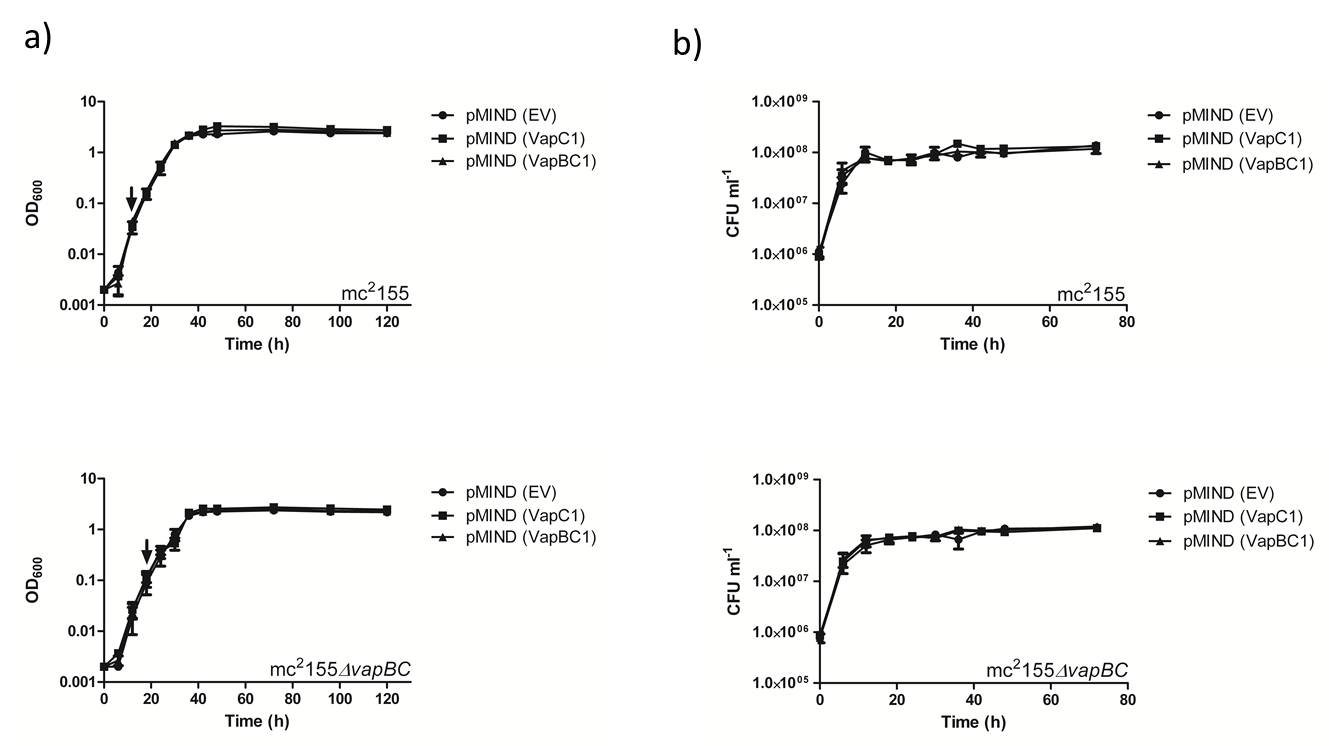


**Figure I. The effect of VapC1 overexpression on the growth and viability of *M. smegmatis* mc^2^155 and ∆*vapBC* strains.** The effect of conditionally expressing VapC1, VapBC1 or empty vector (EV) on growth (a) and cell viability (b) was examined in a wild-type and *vapBC* knockout strain of *M. smegmatis* mc^2^155. Protein expression was induced during early exponential growth (OD_600_ of 0.1-0.2) by the addition of 20 ng.ml^-1^ tetracycline (indicated in a) by black arrow). CFU viability measurements were taken in the 96 hour period following. Data were plotted in Prism V7. Graphs shown are from data acquired from one independent experiment representative of three biological replicates. Results shown indicate the mean ± SD of three technical replicates of each of three biological replicates.
